# Conference Calendar

**DOI:** 10.1002/cfg.276

**Published:** 2003-04


					Comparative and Functional Genomics
Comp Funct Genom 2003; 4: 275-276.
Published online in Wiley InterScience (www.interscience.wiley.com). DOI: 10.1002/cfg.276
Conference Calendar
June-August 2003
Animal models
3rd European Zebrafish Conference: Zebrafish
Genetics and Development: June 11-14
http://zfin.org/zf info/news/mtgs.html#Paris
14th International C. elegans Meeting: June
29-July 3
http://elegans.swmed.edu/Worm meetings.
html
Bacteria
1st FEMS Congress of European
Microbiologists: June 29-July 3
http://www.fems-microbiology.org/
congress2003.htm
GRC -- Archaea: Ecology, Metabolism and
Molecular Biology: August 3-8
http://www.grc.uri.edu/programs/2003/
archaea.htm
Bioinformatics
CHI -- Beyond Genome -- Bioinformatics and
Genome Research: June 16-17
http://www.beyondgenome.com/bin.asp
Intelligent Systems in Molecular Biology (ISMB
2003): June 29-July 3
http://www.iscb.org/ismb2003/index.shtml
IEEE Computer Society Bioinformatics
Conference: August 11-14
http://conferences.computer.org/
Bioinformatics/
GRC -- Bioinformatics: from Inference to
Predictive Models: August 24-29
http://www.grc.uri.edu/programs/2003/bioinf.
htm
Evolution/comparative genomics
Society for Molecular Biology and Evolution
(SMBE): June 26-29
http://smbe2003.bio.uci.edu/
GRC -- Origin Of Life: July 13-18
http://www.grc.uri.edu/programs/2003/origin.
htm
GRC -- Evolutionary and Ecological Functional
Genomics: August 3-8
http://www.grc.uri.edu/programs/2003/
evolecol.htm
General genomics
GRC -- Nucleic Acids: June 1-6
http://www.grc.uri.edu/03sched.htm
CHI -- Beyond Genome: June 16-19
http://www.beyondgenome.com/
XIXth International Congress of Genetics: July
6-11
http://www.geneticscongress2003.com/index.
php
International Congress of Biochemistry and
Molecular Biology (ICBMB 2003): July 20-24
http://www.iubmb2003.org/
Human genome
CSHL -- 68th Symposium: the Genome of
Homo sapiens: May 28-June 2
http://meetings.cshl.org/2003/
2003Symp.htm
GRC -- Human Genetics and Genomics:
August 3-8
http://www.grc.uri.edu/programs/2003/
humangen.htm
Integrative and systems biology
CHI -- Beyond Genome -- Systems Biology:
Pathways to Drug Development: June 18-19
http://www.beyondgenome.com/isb.asp
Metabolomics
GRC -- Enzymes, Co-enzymes and Metabolic
Pathways: July 13-18
http://www.grc.uri.edu/programs/2003/
enzymes.htm
Copyright  2003 John Wiley & Sons, Ltd.
276 Conference Calendar
CHI -- Metabolic Engineering: Autumn/Fall
(dates not yet set)
http://www.healthtech.com/2003/mbp/index.
htm
Pharmacogenomics
GRC -- Toxicogenomics: June 22-27
http://www.grc.uri.edu/programs/2003/toxico.
htm
IBC -- Drug Discovery Technology: August
10-15
http://www.drugdisc.com/section.asp
Plants
14th International Conference on Arabidopsis
Research: June 20-24
http://www.union.wisc.edu/conferenceser
vices/arabidopsis/
7th International Congress on Plant Molecular
Biology: June 23-28
http://www.ispmb2003.com/
ASPB -- Plant Biology 2003: July 26-30
http://www.aspb.org/meetings/pb-2003/
Proteomics
ASMS -- 51st Annual Conference on Mass
Spectrometry and Allied Topics: June 8-12
http://www.asms.org/conferences.php
CHI -- Beyond Genome -- Proteomics:
Applications in Therapeutic and Diagnostic
Development: June 18-19
http://www.beyondgenome.com/pro.asp
GRC -- Proteins: June 22-27
http://www.grc.uri.edu/03sched.htm
6th International Symposium on Mass
Spectrometry in the Health and Life
Sciences -- Molecular and Cellular Proteomics:
August 24-28
http://donatello.ucsf.edu/symposium/default.
htm
Transcriptomics
CHI -- Profiling PCR and Beyond: June 2-6
http://www.healthtech.com/spring.asp
GRC -- Stress-induced Gene Expression: July
27-August 1
http://www.grc.uri.edu/programs/2003/stress.
htm
Yeasts and fungi
European Fission Yeast Meeting (Pombe 2003):
June 27-28
http://pingu.salk.edu/forsburg/pombeweb.
html
XXI International Conference on Yeast
Genetics and Molecular Biology (ICYGMB): July
7-12
http://www.yeast2003.se/
North American/East Coast Fission Yeast
Meeting (US Pombe 2003): July 25-27
http://www.umassmed.edu/pombe2003/
23rd International Specialized Symposium on
Yeasts (ISSY-23): August 26-29
Biotech industry
BIO2003 Annual Convention: June 22-25
http://www.bio.org/events/2003/
Key
ASMS, American Society for Mass Spectrometry:
http://www.asms.org
ASPB, American Society of Plant Biologists:
http://www.aspb.org
CHI, Cambridge Healthtech Institute: http://
www.healthtech.com
CSHL, Cold Spring Harbor Laboratories: http://
meetings.cshl.org/index.htm
EMBL, European Molecular Biology Laboratory:
http//www1.embl-heidelberg.de
ESF IAFG, European Science Foundation -- In-
tegrated Approaches for Functional Genomics,
http://www.functionalgenomics.org.uk/
FEMS, Federation of European Microbiological
Societies: http://www.fems-microbiology.org/
GRC, Gordon Research Conferences: http://
www.grc.uri.edu/
IBC, IBC Conferences: http://www.ibc-lifesci.
com
IEEE, Institute of Electrical and Electronics Engi-
neers, Inc.: http://www.ieee.org/
Copyright  2003 John Wiley & Sons, Ltd. Comp Funct Genom 2003; 4: 275-276.

				

